# A protocol for a randomised controlled trial of prefabricated versus customised foot orthoses for people with rheumatoid arthritis: the FOCOS RA trial [Foot Orthoses – Customised v Off-the-Shelf in Rheumatoid Arthritis]

**DOI:** 10.1186/s13047-018-0272-3

**Published:** 2018-05-31

**Authors:** Kellie S. Gallagher, Jon Godwin, Gordon J. Hendry, Martijn Steultjens, Jim Woodburn

**Affiliations:** 1 University of East London, Stratford, Water Lane, London, England E15 4LZ UK; 2 Glasgow Caledonian University, Institute for Applied Health Research, School of Health & Life Sciences, Cowcaddens Road, Glasgow, Scotland G4 0BA UK

**Keywords:** Rheumatoid arthritis, Orthoses, Cost-effectiveness, Randomised controlled trial

## Abstract

**Background:**

Foot pain is common in rheumatoid arthritis and appears to persist despite modern day medical management. Several clinical practice guidelines currently recommend the use of foot orthoses for the treatment of foot pain in people with rheumatoid arthritis. However, an evidence gap currently exists concerning the comparative clinical- and cost-effectiveness of prefabricated and customised foot orthoses in people with early rheumatoid arthritis. Early intervention with orthotics may offer the best opportunity for positive therapeutic outcomes. The primary aim of this study is to evaluate the comparative clinical- and cost-effectiveness of prefabricated versus customised orthoses for reducing foot pain over 12 months.

**Methods/design:**

This is a multi-centre two-arm parallel randomised controlled trial comparing prefabricated versus customised orthoses in participants with early rheumatoid arthritis (< 2 years disease duration). A total of 160 (a minimum of 80 randomised to each arm) eligible participants will be recruited from United Kingdom National Health Service Rheumatology Outpatient Clinics. The primary outcome will be foot pain measured via the Foot Function Index pain subscale at 12 months. Secondary outcomes will include foot related impairments and disability via the Foot Impact Scale for rheumatoid arthritis, global functional status via the Stanford Health Assessment Questionnaire, foot disease activity via the Rheumatoid Arthritis Foot Disease Activity Index, and health-related quality of life at baseline, 6 and 12 months. Process outcomes will include recruitment/retention rates, data completion rates, intervention adherence rates, and participant intervention and trial participation satisfaction. Cost-utility and cost-effectiveness analyses will be undertaken.

**Discussion:**

Outcome measures collected at baseline, 6 and 12 months will be used to evaluate the comparative clinical- and cost- effectiveness of customised versus prefabricated orthoses for this treatment of early rheumatoid arthritis foot conditions. This trial will help to guide orthotic prescription recommendations for the management of foot pain for people with early rheumatoid arthritis in future.

**Trial registration:**

ISRCTN13654421. Registered 09 February 2016.

## Background

Rheumatoid arthritis (RA) is the commonest form of inflammatory polyarthritis affecting an estimated 645,000 people in the United Kingdom [1–3]. The majority of people with RA will develop foot and ankle problems over the course of their disease that will impact upon their health-related quality of life [1–3]. Large-scale cross-sectional surveys have demonstrated that foot pain and walking disability are commonly experienced by people with RA [4, 5]. In addition, a plethora of mechanistic studies employing gait analysis have demonstrated that people with RA walk slower, with abnormal foot joint rotations and altered plantar pressure loading characteristics [6–8]. Previous research has demonstrated that both mechanical and inflammatory factors contribute to the overall burden of foot disease [8–10]. Accordingly, management strategies for RA-related foot problems include suppression of disease activity with disease-modifying anti-rheumatic, biologic drugs, and/or intra-articular corticosteroids; as well as non-medical management strategies including, therapeutic footwear and foot orthoses (FOs) [11].

Relative to studies of those with established disease, there is a major gap in the current literature concerning the impact and management of foot impairments in early RA. Evidence from cohort studies suggest there is a high prevalence (~ 70%) of foot joint arthritis, pain, and walking disability in patients with early RA (within 2 years of diagnosis) [4, 5, 12]. Functionally important kinematic alterations in foot function consistent with pain avoidance strategies have been detected in participants with early RA via objective analysis of gait [13]. Moreover, recent qualitative research highlights that people with early RA can experience a variety of participation restrictions related to foot impairments [14].

FOs are a recognised non-pharmacological adjunct therapy to standard medical care of people with RA who present with foot and ankle problems. FOs are designed to redistribute load from weight bearing sites such as painful metatarsal phalangeal joints, and to control medial longitudinal arch posture during gait through control of subtalar and midtarsal joint forces [15–18]. Recent systematic reviews and meta-analyses indicate that FOs may be beneficial in reducing pain and forefoot pressures in RA [19, 20]. Guidelines for foot care for people with RA typically recommend the use of FOs in the management of those with relevant foot problems [11, 21–25]. A recent prospective cohort study demonstrated that earlier intervention with FOs for patients with RA predicts favourable foot pain and disability outcomes [12]. This suggests that prescription of FOs targeted earlier may provide the best opportunity for achieving desirable outcomes [12, 26]. However, there have been no randomised controlled trials to evaluate the effectiveness of FOs in early RA.

Prescription practices of FOs for people with RA can be variable between different clinicians, clinics and services, and in the absence of robust evidence these are largely based upon expert opinion and clinical experience [27, 28]. To our knowledge there are currently no treatment guidelines that include specific details regarding the features and types of FOs that provide the greatest therapeutic benefits for people with RA. In clinical practice FOs represent a complex intervention that may or may not include features such as (but not limited to) arch support, forefoot extension (cushioning), and rearfoot control (posting) [27, 29]. There are also a wide variety of materials with different properties available ranging from soft density cushioning devices to rigid supportive devices [27]. Moreover, FOs are often provided as a package of care along with footwear advice, FOs wearing advice, and other foot related co-interventions such as lower limb muscle stretching/strengthening exercises. Historically, the reporting of specific details of the intervention in trials of FOs has generally been poor, however the majority of randomised controlled trials (RCTs) appear to favour FOs with rearfoot posting, forefoot cushioning and firmer density orthotic shells [16, 30, 31].

The literature describes two basic approaches to FO manufacture [32]; i) customised – where FOs are constructed from a patient-specific mould or scan, and ii) prefabricated (also known as ‘off-the-shelf’) – where FOs have been mass- produced from a standard last [33]. In the United Kingdom, standard care tends to involve the use of prefabricated devices unless there is a specific clinical trigger to warrant escalation to customised devices such as localised disease activity affecting the metatarsal heads. In the only direct comparison of customised versus prefabricated FOs in RA to date; modest differences in immediate pressure reduction, pain relief and patient preference for device were reported in favour of customised FOs [19]. Little evidence exists to support one type of device over the other for relief of foot pain or disability in the long term in people with early or established RA. The majority of comparative effectiveness research on customised versus prefabricated FOs has been for relief of pain associated with plantar fasciitis/plantar heel pain, with most studies demonstrating similar clinical effectiveness, but a lower cost with mass-produced prefabricated devices [34–37]. To date, the comparative clinical- and cost- effectiveness of customised versus prefabricated orthoses in early RA has not been tested. A recent study investigated clinical- and cost- effectiveness in established RA [38]. Although this was an exploratory trial with a small sample size, results indicated a decrease of foot pain when using customised FOs compared to simple insoles with no significant cost per quality-adjusted life year gain [38].

An element of customisation can now be undertaken via posting as a form of dose-adjustment and this can be undertaken on both customised (FO shell manufacture from a patient-specific mould/scan) and many newer prefabricated devices. A recent study demonstrated a dose-response effect using various rearfoot wedging increments to access their effect on plantar pressure variables, foot segment rotations and foot joint moments in people who have a pronated foot type [39, 40]. These results support the concept that foot function can be altered by FOs incrementally according to the desired degree of correction at the individual level – a key principle of orthotic dose-adjustment.

Novel orthotic design rules derived from mechanically-based therapeutic targets [7] have recently been used in a lab-based mechanistic study to inform the manufacture of enhanced customised FOs, versus traditionally manufactured customised FOs in people with early RA who had passively correctable pes planovalgus [41]. While no long-term outcome data was evaluated, superior immediate mode-of-action determined by gait analysis, and better patient experience in terms of self-reported comfort and fit were recorded for highly personalised devices [41]. Further research is required to determine whether or not customised FOs result in significant clinical benefits in order to justify the additional costs of manufacture.

This study is designed to address an important gap in the research literature concerning the comparative clinical- and cost-effectiveness of customised versus prefabricated FOs in early RA. Specifically, the primary aim of this study is to evaluate the comparative clinical- and cost-effectiveness of prefabricated versus customised FOs at reducing foot pain over 12 months. The secondary aims include the following:-

i) to evaluate the effectiveness, in terms of all improvements in secondary outcomes including foot-related disability, Foot Function Index (FFI) and Foot Impact Scale (FIS) subscales, localised foot disease activity (RADAI-F5), global disability (HAQ), and health related quality of life (EQ5D 5 L) achieved at 6 and 12 months following intervention with either prefabricated or customised FOs;
ii) to evaluate the effectiveness in terms of overall patient satisfaction (as measured by a participant satisfaction questionnaire);
iii) to evaluate the cost-effectiveness of prefabricated in comparison with customised FOs;
iv) to explore patient opinions, perceptions, and experiences of benefit following orthotic therapy (prospective serial interviews and patient satisfaction questionnaire).

## Methods/design

### Study design and setting

**Fig. 1:**
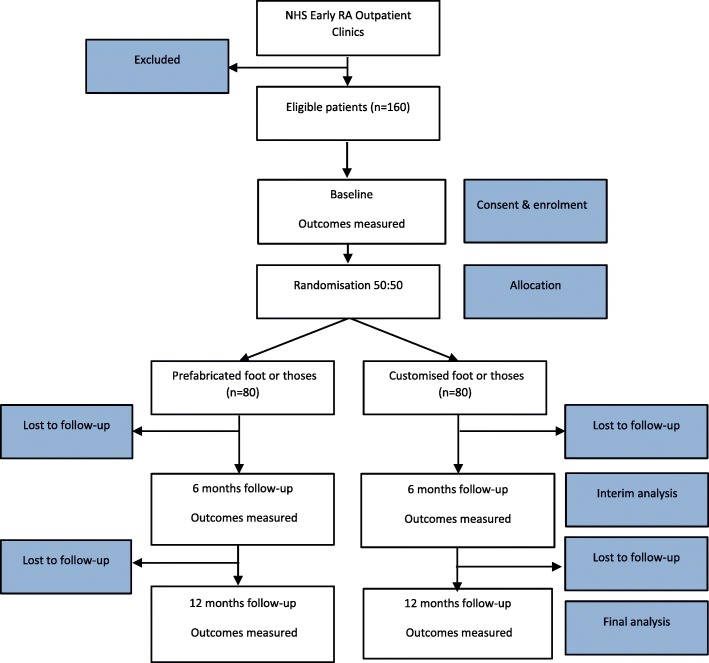
CONSORT flow diagram outlining the flow of participants through the trial

This trial is designed as a multi-centre two-arm parallel RCT. A CONSORT flow diagram is presented (Fig. 1), which outlines the flow of participants through the trial. Participants will be identified by their Rheumatologist within the early RA clinics in National Health Services Grampian, Fife, Lanarkshire, Lothian Health Boards (Scotland), as well as Dorset Healthcare University Trust, and Homerton University Hospital (England). Each site will have an appointed independent outcome assessor (either a rheumatology nurse specialist or a research nurse) who has received trial protocol training, and at least one United Kingdom registered Health Care Professionals Council podiatrist who has either musculoskeletal or rheumatology experience working at least at an National Health Service band 6 level and who has received trial specific intervention delivery training. The intervention will be supplied by the podiatrist and the outcomes will be collected by the outcome assessor at each site over three time points: baseline, and 6 and 12 months from baseline. Recruitment commenced May 2016 and the study end (final follow-up, final participant) is anticipated by 30th April 2019.

### Eligibility criteria

Participants will be eligible for inclusion if they are aged > 18 years and diagnosed with RA < 2 years previously based on the 2010 ACR/EULAR classification criteria [42]. Participants will be eligible if they meet the minimum threshold score of ≥20 mm on a visual analogue scale for foot pain which is localised to any one of the following: metatarsal phalangeal joints, midfoot, rearfoot, and/or periarticular tendons surrounding the ankle/subtalar joints. If any rearfoot or forefoot bony deformity or malalignment is present, this must be passively correctable as tested through a range of motion assessment. Participants must not have worn FOs in the previous 6 weeks if provided prior to RA diagnosis.

Participants will be excluded if they had been diagnosed with any neurological or endocrine diseases such as diabetes, which could potentially affect peripheral nerves, foot structure, function and pain perception. Additionally, exclusions will occur if they have had any trauma or injury affecting the musculoskeletal systems of the lower limb of foot.

### Interventions

Both FO interventions (customised versus prefabricated) represent complex interventions that are comprised of several components. Given the wide variety of FO prescription practices, the authors have undertaken a partially pragmatic approach to the FO interventions whereby customisation based on participant characteristics and driven by clinical design rules will be permitted with certain restrictions to avoid prescription variation. Clinical design rules will guide clinicians on prescriptions based on participant’s foot characteristics, for example the presence of forefoot pain would trigger addition of forefoot cushioning to the device. Delivery of co-interventions such as footwear advice will also be permitted and will be monitored for the duration of the study.

### Prefabricated foot orthoses

**Fig. 2:**
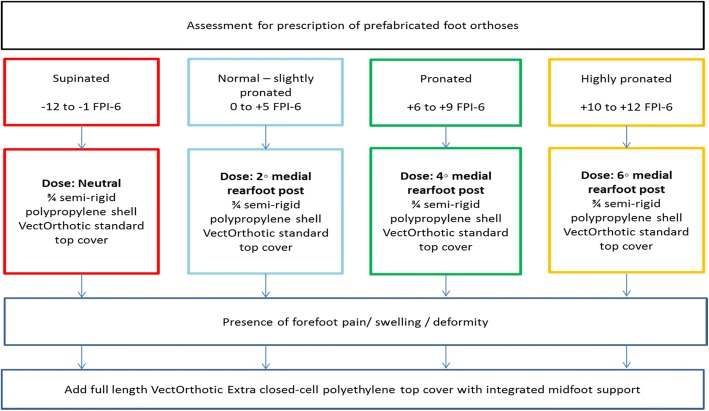
Summary of prefabricated foot orthoses prescription protocol

This trial will use an ‘off-the-shelf’ device (VectOrthotic®, Healthy Step United Kingdom) for the prefabricated FOs intervention arm. This device is manufactured from semi-rigid polypropylene and dose (posting) can be adjusted according to individual participant characteristics. The VectOrthotic device is accompanied by “click-in” rearfoot posts in 2°, 4° and 6° degree doses, as well as adhesive full length top covers. The VectOrthotic has been used previously as an intervention in an exploratory study of adults with mechanical foot pain and their use was associated with a decrease in foot pain relative to sham orthoses [43]. Extrinsic rearfoot posting will be provided to correct foot posture according to the static foot posture measurements using the Foot Posture Index [44, 45]. Rearfoot posting will be either 0°, 2°, 4° or 6° depending on the degree of supinated to pronated foot posture. All devices will be ¾ length with a VectOrthotic® standard top cover (unless additional forefoot protection is specifically required due to the presence of forefoot pain, swelling and/or deformity). Should additional forefoot protection be required, the VectOrthotic® Extra closed-cell polyethylene top cover (with integrated midfoot support) extending to the toes will be added to the prescription. The prescription flow chart is outlined (Fig. 2).

### Customised foot orthoses

Participants randomised to this group will receive customised FOs for both feet. The customised shell will be obtained from foam box impressions of both feet using the functional semi-weight-bearing subtalar joint neutral position method [46]. The participant will be positioned in a chair with the hip and knee joints at 90° flexion. The foot will then be placed on the foam box, and the podiatrist will press the participant’s foot down until the bottom of the box is reached while maintaining the foot sole parallel to the floor and the rearfoot aligned in the subtalar neutral position [47]. Hand pressure will be applied to the dorsum of the midfoot to prevent supination of the midtarsal joint due to the reactive force of the foam.

The basic contour of the orthotic shell will be based on the description of the modified Root style of orthosis, which is considered to be the most commonly prescribed type of customised orthotic in podiatric practice [48]. The orthotic will be manufactured from semi-rigid polypropylene and posted according to information obtained from assessment of foot posture (using the Foot Posture Index). The shell will be modified to include a deep heel cup (18 mm) and medial flange [49]. The customised FOs will be manufactured by a commercial laboratory (Firefly Custom Made Foot Orthoses, Sligo, Ireland). Extrinsic rearfoot posting will be provided to correct foot posture according to the static foot posture measurements using the Foot Posture Index to correct the rearfoot posture to neutral (calcaneal vertical) using cast forefoot balancing techniques [50]. This will allow greater design freedom through rearfoot posture correction to a precision of the nearest 1°.

**Fig. 3:**
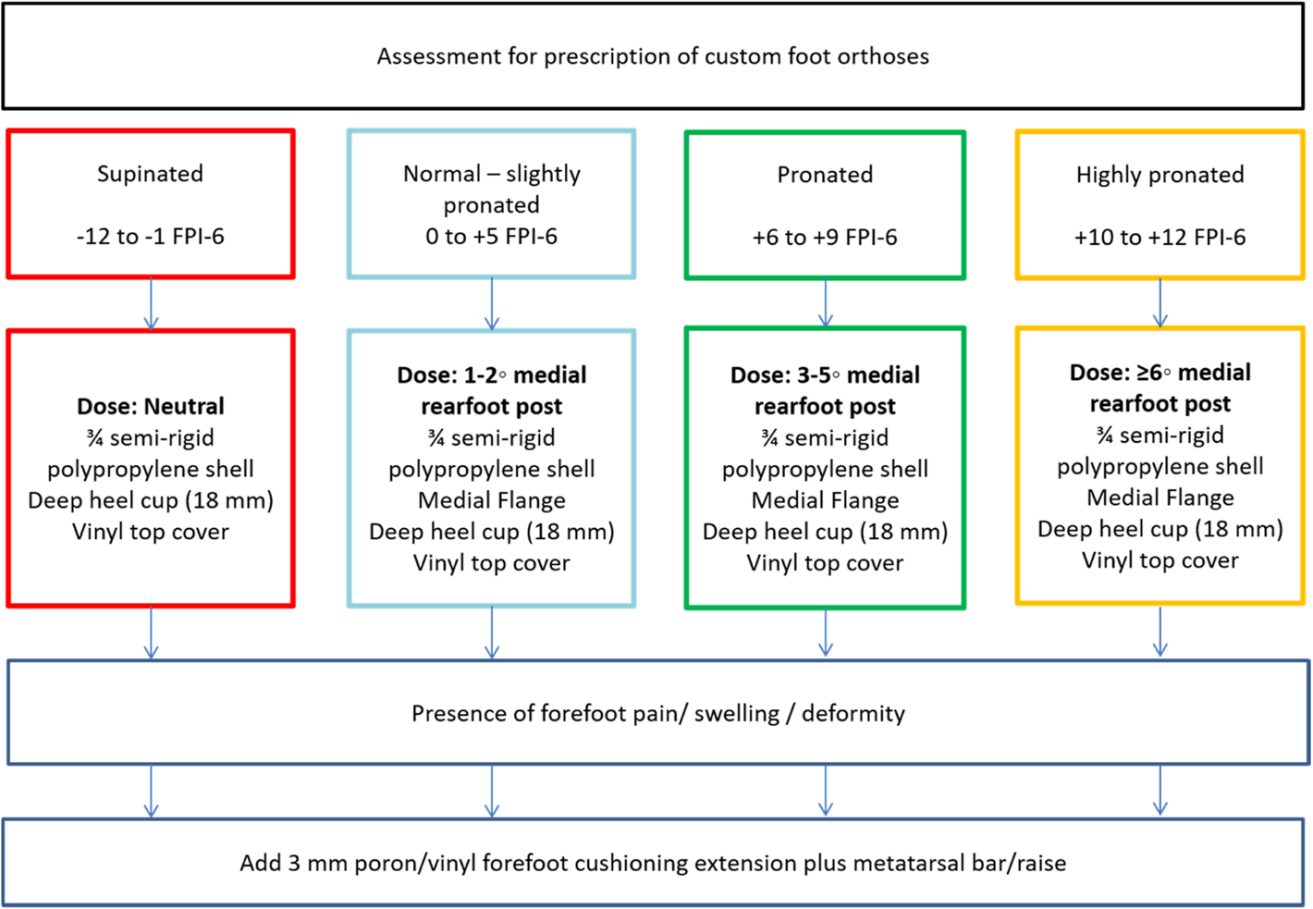
Summary of customised foot orthoses prescription protocol

The prescription flowchart for this intervention arm is outlined (Fig. 3). All devices will be ¾ length with a vinyl top cover (unless forefoot cushioning is specifically required). Should cushioning be required, 3 mm poron/vinyl top cover extending to the toes with an integrated metatarsal raise will be added to the prescription for manufacture.

### Study procedures

An overview of study procedure is outlined in the SPIRIT table (Table 1). For both intervention arms, each participant will receive a minimum of 2 one-to-one sessions with the podiatrist pertaining to the FOs interventions:-.

**Table 1:** SPIRIT table for study procedure

	Pre-study screening/consent	Baseline	Randomisation /Clinical visit 1	Clinical visit 2	Clinical visit 3	6 months follow up	12 months follow up
	−1	0	T1^a^	T2^b^	T3	F1	F2
Enrolment							
Eligibility screen	X						
Informed consent	X						
Allocation			X				
Measurements							
FFI pain		X				X	X
FFI disability		X				X	X
FFI functional limitation		X				X	X
FIS-RA		X				X	X
EQ-5D-5 L		X				X	X
HAQ		X				X	X
DAS-28		X				X	X
CSRI						X	X
Interventions							
Customised foot orthoses			X	X	X		
Prefabricated foot orthoses			X		X		

*FFI* Foot function index, *FIS-RA* Foot impact scale for rheumatoid arthritis, *HAQ* Health assessment questionnaire, *DAS-28* disease activity score 28 joints, *CSRI* Client service receipt inventory

T1^a^ randomisation was triggered by the intervention clinician at the first clinical visit using the online system

T2^b^for customised FO group, manufacture required an additional fitting appointment approximately 2 weeks following clinical visit 1

#### Session 1

All participants will be assessed in order to inform their FOs prescription. During this session participants will receive standard podiatry co-interventions. In the prefabricated FOs arm only, participants will receive their FOs at this session following the assessment by the podiatrist (this is in line with routine clinical practice for prefabricated FOs – which can be provided ‘off-the-shelf’ on the same day). The podiatrist will check fit-to-feet, and fit-to-shoe or the orthotic, and will seek subjective information from the participant concerning initial comfort and fit [41].

#### Session 2 [custom arm only]

Participants in the customised FOs arm a will return for the fitting of either their customised FOs within 2–3 weeks of their session 1 appointment (this is in line with routine clinical practice where there is a gap between initial assessment and fitting to allow for manufacture of the customised device). At fitting stage, the podiatrist will check fit-to-feet, and fit-to-shoe or the orthotic, and will seek subjective information from the participant concerning initial comfort and fit [41].

#### Session 3

All participants will return for a review of their orthotic device 6–8 weeks after initial fitting of the respective FO device at either session 1 (prefabricated) or session 2 (customised). At this appointment the podiatrist will repeat the initial assessments that led to the prescription to ensure the orthotic device is still appropriate for each participant. The podiatrist will review subjective information from the participant concerning comfort, fit, and self-reported efficacy, including whether or not there has been any change in symptoms and/or short-term benefit over the previous 6–8 weeks using Visual Analogue and Likert Scales.

#### Unscheduled foot orthoses review sessions

From initial fitting of FOs to the end of the trial period at 12 months from baseline, self-referral for review of FOs will be permitted for participants in either treatment arm where there are adverse reactions, and/or loss of or damage to foot orthoses. To facilitate this, participants will be provided with the contact details for their podiatrist in order to book unscheduled FOs review sessions.

### Study outcome measures

The primary outcome measure will be foot pain, which will be measured at 12 months using the Foot Function Index pain subscale (FFI_pain_), which is a composite score for foot pain [51]. The FFI is a widely used, valid and reliable self-administered questionnaire consisting of 23 items grouped into 3 domains: foot pain (9 items), disability (9 items), and activity limitation (5 items) [51]. At present, the FFI is one of the few instruments for scoring foot-related disability that has been rated positively for responsiveness-to-change [6] and has been employed frequently as a primary outcome measure in several previous RCTs of FOs for people with RA [16, 52, 53]. For each subscale, items are rated using a 100 mm Visual Analogue Scale, and a composite score is calculated by summing items and dividing by the total number of items in that subscale. As outlined in the original scoring system [51] any item marked as not applicable will be excluded from the calculation of the total possible score. A higher score is indicative of more severe foot pain and disability.

### Secondary outcome measures

The remaining FFI subscales for disability (FFI_dis_) and functional limitation (FFI_fl_) will be measured as secondary outcomes. In addition, the Foot Impact Scale for RA (FIS_RA_) will be adopted as a secondary outcome measure to provide a disease-specific measurement of localised disease impact [54]. This is a 51-item questionnaire with dichotomous scoring system (true/not true responses) and two subscales for impairment/footwear and activity limitation/participation restriction. Responses in the true column are summated to give a total score for each subscale.

The EQ-5D-5 L [55] is a valid and reliable measure of health-related quality of life in adults and will be used primarily for the purposes of the cost-utility analysis aspect of the embedded health economic evaluation of the intervention. The EQ-5D-5 L is a 5-item questionnaire that requires a response on a 5-point Likert scale, and responses will be used to calculate quality-adjusted life years gained.

A satisfaction questionnaire consisting of numerical rating and Likert scales will be used to measure orthotic device comfort, fit, and self-reported efficacy symptoms and activity levels. Participant reporting of adverse events will also be permitted to provide further comments using open-ended responses within this questionnaire. In addition, a small random sample of participants will be invited to take part in an interview to explore experiences of the interventions and perceptions of improved/deteriorated outcomes.

Global disability will be measured using the Health Assessment Questionnaire [56]. Global disease activity will be measured using the Disease Activity Score using 28 joints [57]. Disease duration will be recorded as the time in months from onset of symptoms and time in months from disease diagnosis as self-reported by the participant.

### Health economic evaluation

The health economic analysis will conform to a superiority trial analysis and will address both cost-effectiveness (cost per unit of improvement of the primary outcome – the FFIpain) and cost utility using quality-adjusted life years measured using the EQ-5D-5 L.

Costs of treatment in either arm will be considered from a societal perspective. For prefabricated FOs, the costs collected will include the unit cost of each prefabricated device prescribed, and staff time for assessments and review appointments. Similarly for customised FOs, the costs collected will include costs associated with manufacture of FOs including materials and staff time, as well as staff time for assessments and review appointments. In addition, the costs of systemic therapies in terms of biological agents, disease-modifying anti-rheumatic drugs and non-steroidal anti-inflammatories will be recorded for both trial arms.

For both trial arms and in addition to FOs related costs, we will record the use of foot care services throughout the trial. These will be costed using National Health Service pay and prices or, where appropriate, using other (e.g. shop or internet bought) sources. Out of pocket expenses for over the counter products, and complementary and alternative therapies, as well as travel to/from health appointments and time off work will also be included. All cost data will be collected using a combination of participant medical records, and an adapted version of the Client Service Receipt Inventory [58] resource use checklist. Data on programme costs and those on further impacts will be aggregated and the statistical significance of differences in cost per patient between trial arms assessed by appropriate methods depending on the distributional characteristics of the data.

### Sample size

In the absence of availability of detailed information on the clinical meaningful difference in outcome measured using the FFI pain subscale, the calculation of our desired effect size was based upon previous findings from an FOs trial for people with RA [52]. This trial evaluated 6 month FFI pain subscale scores at 6 months following intervention with either a functional FO versus a non-functional placebo-type flat FO. Mean difference at 6-month outcome (9.93) and pooled standard deviation (19.77) values were obtained from this study to calculate a standardised mean difference. For a two-tailed hypothesis, to detect a standardised mean difference of 0.5 (9.93/19.77) (a medium to large effect size) at 0.05 significance, 0.8 power and 20% attrition (based on the randomised controlled trials by Woodburn et al. [30]), we require 160 participants (80 per arm) [Statistics Calculators, 2015].

### Recruitment

Participants meeting the selection criteria will be recruited from ‘early arthritis’ rheumatology outpatients’ clinics within the National Health Service Health Boards/Trusts of the participating centres.

### Randomisation

Randomisation will be conducted via permuted block. This means there will be variable size blocks with allocation to customised (a) or prefabricated (b) for 160 participants. In order to conceal the process from local trial site personnel, this will be administered via web randomisation. Permuted block randomisation will be performed using a bespoke Fortran program with an intrinsic pseudo-random number generator and the resulting table will be accessed by a bespoke Fortran web application on a secure server, to provide and log allocations sequentially on-line as required. Each intervention podiatrist will have access to a web ‘link’ which will randomly allocate the participants into each arm. This will be undertaken by the intervention podiatrists once they have concluded their respective foot assessments.

### Allocation concealment

Allocation will be conducted once eligibility checks have been performed and only once the participant has been registered in the trial. The central randomisation administrator who will follow the web based allocation will be masked to the identity of participants and all primary and secondary outcomes.

### Blinding/masking

The nature of this trial dictates that blinding of the clinicians delivering interventions and the participants is not possible. However the outcome assessor will be blind to group allocation, and will not be involved in any delivery of therapeutic interventions. Participants will be instructed not to disclose their group allocation to the outcome assessor. Any breaches in blinding protocol will be recorded.

### Analysis

Data will be analysed according to the intention-to-treat principle. Data will be explored to determine whether or not potential confounders need to be accounted for in the final analysis. Should we detect any imbalances between intervention groups, we expect that the analysis of the primary outcome (FFI pain subscale) will be conducted using an analysis of covariance (ANCOVA) with adjustment for relevant baseline values and potential confounders. For example, to account for systemic disease activity as a covariate, the FFI pain subscale change scores will be adjusted according to both FFI pain subscale baseline scores and the change scores of the Disease Activity Scale for 28 joints between baseline and follow up. Statistical methods for secondary/additional analyses: Similarly, for comparison of all other secondary outcomes, between group scores from baseline to 12 months will be compared using ANCOVA with adjustment for baseline values and potential confounders. All estimates for primary and secondary outcomes will be reported with estimated effect sizes alongside 95% confidence intervals.

### Health economics analyses

Outcomes will be assessed using the primary trial outcome and the EQ-5D-5 L. The primary analysis will be undertaken at 12 months from an National Health Service and Personal Social Services perspective. A broader perspective including patient’s personal expenditures will be included in a sensitivity analysis. Incremental cost effectiveness ratios will be computed by comparing the costs and outcomes of both arms of the trial. The difference in effectiveness will be expressed in terms of the change in score on the primary outcome measure (cost-effectiveness analysis). The difference in utility will be expressed in terms of quality adjusted life years calculated using patient reported EQ-5D 5 L data. This will be used in a cost utility analysis to calculate the incremental cost per quality adjusted life years gained.

### Missing data

For missing data (participant withdrawal or interim missing data), the plausibility of missing data models ‘missing at random’, ‘missing completely at random’, ‘missing not at random’ will be ranked by the trial personnel (informed by the available trial data). The most plausible missing data model will be selected and a sensitivity analysis will be conducted accordingly to explore the effect of departures from assumptions made in the primary analysis. Upon completion of sensitivity analyses, an appropriate method of missing data imputation will be selected.

### Ethical consideration

Ethical approval for this study has been obtained by the East of England – Essex Research Ethics Committee Ref: 15/EE/0410. Registration of this RCT has been completed with the ISRCTN registry: ISRCTN31652.

## Discussion

FOs have been used clinically for many decades for the treatment of foot pain in patients with RA, although their effectiveness has not been rigorously evaluated as a management method. Despite this, foot care recommendations feature in many United Kingdom and European clinical guidelines. However, the level of supporting evidence is low, mainly at ‘good clinical practice’ and ‘expert opinion’ agreement level [26]. This could be due to various reasons: the numerous types of FOs available in the market place; the variation of prescription habits between clinicians; and the biomechanical effects of the FOs on the lower limb, which we believe to be clinically beneficial are limited throughout the literature [59]. Further, customised orthoses commonly follow the ‘subtalar joint neutral theory’ for foot morphology even when criticised for its reliability and validity [60]. Three reviews investigating the effect of FOs in RA patients report a general consensus that FOs are beneficial [61–63], yet no insight is given into prescription guidelines or treatment recommendations.

This trial protocol has been designed to provide robust results on orthotic treatment and guidelines in an RA cohort. This is a randomised trial which includes concealed allocation using a web based system, blinding of outcome assessors, blinded data analysis and the use of outcome measures with proven reliability and validity in pain, satisfaction, disease activity and functional limitation. Further, all podiatrists will be trained and provided with prescription protocols (Figs. 2 and 3) to follow ensuring similar prescriptions between the 6 recruitment sites.

This trial aims to provide clinically relevant and robust evidence in regards to clinical outcomes, patient satisfaction and cost-effectiveness. Each of the research questions that will be investigated in this study will provide National Health Services with further knowledge about the use of FOs in this selected patient cohort. Clinical effectiveness evaluation will provide evidence for the National Health Service to practice around the highest standards as well as being aware of patient satisfaction. We anticipate that this project will provide vital evidence and thus guidance to inform clinical decision making in future.

## Abbreviations

ACR: American College of Rheumatology
ANCOVA: Analysis of covariance
EULAR: European League Against Rheumatism
FFI: Foot Function Index
FFI_dis_: Foot Function Index for disability
FFI_fl_: Foot Function Index for functional limitation
FFI_pain_: Foot Function Index pain subscale
FIS: Foot Impact Scale
FIS_RA_: Foot Impact Scale for Rheumatoid Arthritis
FO: Foot Orthoses
HAQ: Health Assessment Questionnaire
HRQoL: Health Related Quality of Life
RA: Rheumatoid Arthritis
RADAI-F5: Rheumatoid Arthritis foot Disease Activity Index
RCT: Randomised Controlled Trial
